# A comparison of absolute performance of different correlative and mechanistic species distribution models in an independent area

**DOI:** 10.1002/ece3.2332

**Published:** 2016-07-27

**Authors:** Farzin Shabani, Lalit Kumar, Mohsen Ahmadi

**Affiliations:** ^1^ Ecosystem ManagementSchool of Environmental and Rural ScienceUniversity of New EnglandArmidaleNSW2351Australia; ^2^ Department of Natural ResourcesIsfahan University of TechnologyIsfahanIran

**Keywords:** Bioclimatic model, correlative model, fundamental niche, mechanistic niche model, modeling methods, realized niche, species distribution model

## Abstract

To investigate the comparative abilities of six different bioclimatic models in an independent area, utilizing the distribution of eight different species available at a global scale and in Australia. Global scale and Australia. We tested a variety of bioclimatic models for eight different plant species employing five discriminatory correlative species distribution models (SDMs) including Generalized Linear Model (GLM), MaxEnt, Random Forest (RF), Boosted Regression Tree (BRT), Bioclim, together with CLIMEX (CL) as a mechanistic niche model. These models were fitted using a training dataset of available global data, but with the exclusion of Australian locations. The capabilities of these techniques in projecting suitable climate, based on independent records for these species in Australia, were compared. Thus, Australia is not used to calibrate the models and therefore it is as an independent area regarding geographic locations. To assess and compare performance, we utilized the area under the receiver operating characteristic (ROC) curves (AUC), true skill statistic (TSS), and fractional predicted areas for all SDMs. In addition, we assessed satisfactory agreements between the outputs of the six different bioclimatic models, for all eight species in Australia. The modeling method impacted on potential distribution predictions under current climate. However, the utilization of sensitivity and the fractional predicted areas showed that GLM, MaxEnt, Bioclim, and CL had the highest sensitivity for Australian climate conditions. Bioclim calculated the highest fractional predicted area of an independent area, while RF and BRT were poor. For many applications, it is difficult to decide which bioclimatic model to use. This research shows that variable results are obtained using different SDMs in an independent area. This research also shows that the SDMs produce different results for different species; for example, Bioclim may not be good for one species but works better for other species. Also, when projecting a “large” number of species into novel environments or in an independent area, the selection of the “best” model/technique is often less reliable than an ensemble modeling approach. In addition, it is vital to understand the accuracy of SDMs' predictions. Further, while TSS, together with fractional predicted areas, are appropriate tools for the measurement of accuracy between model results, particularly when undertaking projections on an independent area, AUC has been proved not to be. Our study highlights that each one of these models (CL, Bioclim, GLM, MaxEnt, BRT, and RF) provides slightly different results on projections and that it may be safer to use an ensemble of models.

## Introduction

Species distribution models (SDMs) combine empirical data on the occurrences or abundance of a species with data on related environmental factors. Such models are used to predict distributions across landscapes and to gather new insights into ecological and evolutionary development, sometimes dependent upon extrapolation in time and space, and are widely used in terrestrial, marine, and freshwater applications. Methodological differences in specific discipline applications reflect differences in the mobility of species and in practices. The realism and robustness of the model are dependent on the selection and relevancy of predictors, method, scale, interaction of geographic and environmental factors, extent of model calibration, and levels of projection (i.e., inter‐ or extrapolation). Present links between modeling practices and ecological sciences are generally poor, which limits development in the field. Challenges that remain include refining presence‐only data modeling methods, model selection and evaluation, the handling of biotic interactions, and the measurement of model uncertainty (Elith and Leathwick 2009). While an understanding of the effects on the patterns of a species distribution in novel climates is vital in the management and planning of conservation, there is a high uncertainty in the projection of future ecological scenarios. Studies on the modeling of the ranges of species have encountered conceptual, theoretical, and methodological difficulties, leading to problems in interpreting results, problematic in the case of both current and future modeled environments (Dormann 2007; Webber et al. 2011; Vicente et al. 2014).

The bioclimatic models most frequently used are correlative, in that through the incorporation of statistical or machine learning techniques they integrate readily available distribution records of species with spatial environmental data (Elith and Leathwick 2009). A more lengthy and data‐intensive alternative method is to link the species ecophysiological responses to environmental variables in mechanistic bioclimatic models (Kearney and Porter 2009). The framing of the research question is a guide to which components of a species niche will be represented in a particular modeling technique (Venette et al. 2010; Watling et al. 2015), the most applicable method of modeling and what training data should be used (Soberón and Nakamura 2009). Such selections, in turn, influence the actual model projections. Novel climates are paramount in the ecology of invasive species under changing climate and in the formulation of management policy frameworks from such studies. Climatic factors, biotic interactions, and species dispersal are the three most basic determinants of the range of a species (Soberón and Nakamura 2009). The outcomes of bioclimatic modeling exploring habitat suitability under novel climates should approximate the Grinnellian fundamental niche (Soberón 2007). The absolute minimum requirement is that they should represent the realized Hutchinsonian niche (Soberón 2007) which underlies the native range of that species.

The scientific expertise and essential resources required for the parameterization of mechanistic models are as yet unavailable for many species, limiting application to higher‐profile species (Shabani et al. 2015). Alternatively, correlative models can be parameterized quickly and have minimal essential requirements, using readily available distribution records and spatial environmental data. This virtually assures the continuation of their general usage. Some authors have raised concerns regarding extrapolation problems (Sutherst and Bourne 2009), arguing for a more cautious critical evaluation of correlative model performance in an independent area in order to identify and resolve such problems (Rodda et al. 2011).

In modeling work, there is much uncertainty about the selection of the appropriate model to use. There are a variety of models available; each one of them functions slightly differently and needs slightly different background data. Therefore, for the layman, it is difficult to decide which one is the best for their particular application. Thus, it is highly important to compare a number of models across a large number of different species because models perform differently based on different species and distributions. In this study, primary modeling focused on building General Linear Model (GLM), MaxEnt, Bioclim, Random Forest (RF), Boosted Regression Tree (BRT), and CLIMEX (CL) models illustrating the climate suitability for *Asparagus asparagoides*,* Triticum aestivum* L., *Lantana camara* L.*, Opuntia robusta*,* Triadica sebifera*,* Fusarium oxysporum* f. spp., *Phoenix dactylifera* L., and *Gossypium* (cotton) by utilizing a global‐scale training dataset, excluding Australian distribution records. Thereafter, in order to evaluate the possibilities of SDMs, we made a comparison of the power of these six modeling techniques in the projection of suitable climate, using observed records of distributions of the eight species in Australia. Finally, we explored the combination of the correlative and mechanistic modeling in complementary fashion, as a means to developing a more robust technique for bioclimatic modeling.

## Materials and Methods

### Distribution records

Our species distribution data were compiled from a number of sources. Global distribution data were taken from the Global Biodiversity Information Facility (2015), Atlas of Living Australia (2014), as well as scientific literature. ENMTools (Warren et al. 2010) was utilized in making the georeferenced occurrence data of each grid cell equal 1. In other words, the existence of multiple records in a single grid cell has no bearing on the projections or statistical performance testing of the models. The global, Australian, and the modified distribution records of all eight species are summarized in Table 1. The dataset includes the total of both native and exotic distribution records (Shabani and Kumar 2015), as it was beyond the study scope to differentiate the effect of including only native, exotic, or both, on the abilities of techniques to project climate suitability.

**Table 1:** The known distribution records of eight species at global scale, Australia and the modified numbers of records in Australia through ENMTools

Dataset	*Asparagus asparagoides*	*Phoenix dactylifera* L.	*Fusarium oxysporum* f. spp	*Gossypium*	*Lantana camara* L.	*Opuntia robusta*	*Triadica sebifera*	*Triticum aestivum* L.
Global scale	4924	529	230	17,322	17,856	299	1724	50,337
Australia	3836	51	30	2656	8324	57	53	142

### Species distribution modeling

Six bioclimatic models were applied to the eight chosen species: for correlative modeling, the GLM (McCullagh and Nelder 1989), a simple regression‐based method, was used together with three machine learning algorithms including Maximum Entropy (MaxEnt) (Phillips et al. 2006), BRT (Elith et al. 2008), and RF (Breiman 2001). We also considered Bioclim (Busby 1991) as the most commonly used envelope procedure and CL, a mechanistic niche model. Construction included a training dataset, which included all available global data, but excluded Australian distribution records. All models used 10' resolution historical data (1975H) available at CliMond database (Kriticos et al. 2012), with intermodel statistical comparison limited to independent distribution records in Australia.

### Correlative models

#### Generalized Linear Model

In GLM, the iterative weighted linear regression technique was used to arrive at the estimated maximum likelihood of the parameters, with observations distributed in terms of an exponential family and systematic effects made linear by suitable transformation. For GLM, parametric functions were employed to link the variable of response to a combination of linear and quadratic explanatory variables. The GLMs were fitted with a standard polynomial approach together with an automatic stepwise model selection based on the Akaike information criterion.

#### MAXENT

For MaxEnt model, we utilized MaxEnt desktop version 3.3.3k (Phillips et al. 2006) with modified parameters (Phillips and Dudík 2008). MaxEnt is reliant on a geographical background (Guillera‐Arroita et al. 2014), defined by the user for the purpose of comparing the climate of a sampled reference set of grid cells with that of the grid cells in which the species is found to be present. The background dataset definition influences the model results significantly (Elith et al. 2011) and should include the species full environmental range of those areas that have been searched (Elith et al. 2010). In MaxEnt algorithm, we compared the complex interactions between presence locations and variables to similar interactions with background locations, to establish the maximum entropy probability distribution closest to uniform, subject to constraints imposed by the spatial distributions observed and environmental factors. By minimizing relative entropy between data of known location and background point data in such a manner, maximum entropy probability distribution is optimized (Phillips et al. 2006).

#### Bioclim

Bioclim (as well as GLM, MaxEnt, BRT, and RF) adopts the principle that current distribution is the foremost indicator of the climatic requirements of a species, to correlate the climate variables in observed distributions. Thus, such a correlative model uses the realized niche to describe bioclimatic envelopes; in that realistically observed distributions are limited by nonclimatic factors, inclusive of biotic interactions. Alternative bioclimatic models seek a mechanistic relationship between climatic parameters and species response with a more physiological basis (Woodward 1987; Pearson and Dawson 2003). Such models identify the fundamental niche through the modeling of the physiological limiting mechanisms of the climatic requirements of the species. Some of the criticism of bioclimatic modeling is that the biotic interactions, species dispersal, and changes through evolution are not integrated into the modeling process. It should be noted that biotic interactions, physical limitations to dispersal, and the impacts of human intrusions prove that realized niches, as utilized in methodologies of correlative Bioclimate envelopes, may not signify the absolute limits of ranges and that thus, a future distribution may be based on a very different realized niche (Pearson and Dawson 2003). Therefore, Bioclim or the environmental envelope model refers to the “climate profile” of a species based on a “boxcar” or “parallelepiped classifier” (Busby 1991). This simple hyperbox classifier defines species potential range as the multidimensional environmental space bounded by the minimum and maximum values for all presences (or 95% of them, or other similar variations). As we aimed to extrapolate the prediction over an independent area, we parameterized Bioclim based on the outlier‐corrected (Skov and Svenning 2004) minimum and maximum observed values at species presences for each climatic variable as it provides lesser conservative results. To compute each species potential distribution, we employed Bioclim in the “Dismo” package (Hijmans and Elith 2015).

#### Random Forest

Random Forest is one of the most precise‐in‐performance of classification or regression tree‐based models, in which the use of bootstrap aggregation selects many subsamples from the data, and through a bagging algorithm generates a large number of decorrelated regression trees (Breiman 2001). Thus, RF combines tree predictors in a manner that each tree is dependent on independently sampled random vector values, with similar distribution for all trees in the forest (Breiman 2001). Thus, we utilized RF because it forms a grouping of unpruned classification or regression trees, constructed using training data bootstrap samples and random feature selection in the induction of the tree. An aggregating (majority vote or averaging) of the predictions of the ensemble facilitates the prediction (Svetnik et al. 2003). The out‐of‐bag observations in each tree are used to predict model errors and variables' importance. Finally, similar to an ensemble approach, the predictions from grown decision trees are averaged. In our study, the “RandomForest” package was used (Liaw and Wiener 2002) in fitting the RF models.

#### Boosted Regression Tree

Using an identical background area to the MaxEnt modeling and all eight species, in BRT model, we fitted a large number of combinations (i.e., decision trees) iteratively and combined them to an optimal model to improve the performance of prediction. BRT uses two multiple regression tree algorithms (by a binary division of predictor space into rectangles, it relates predictor responses to establish expanses with the most homogeneous responses to predictors) and boosting (an added procedure, merging fitted trees for greater modeling accuracy). BRT was fitted using the “GBM” package (Ridgeway 2006) in R environment v 3.1.2 (R Development Core Team, 2014) with additional setting code recommended by Elith et al. (2008).

### Bioclim variables, background data, and the methods for providing weights for species records

Through the jackknife analysis method and the correlation of coefficient results by Pearson correlation technique for each species, the most important variables with low correlation (*R* ^2^ < 0.5) were selected and used in all correlative modeling approaches. For example, bio1 (Annual mean temperature (°C)), bio3 (Isothermality), bio8 (Mean temperature of wettest quarter (°C)), bio12 (Annual precipitation (mm)), bio15 (Precipitation seasonality (C of V)), bio17 (Precipitation of driest quarter (mm)), bio20 (Annual mean radiation (W·m^−2^)), bio21 (Highest weekly radiation (W·m^−2^), bio24 (Radiation of wettest quarter (W·m^−2^)), bio31 (Moisture index seasonality (C of V)), bio34 (Mean moisture index of warmest quarter), and bio35 (Mean moisture index of coldest quarter) were selected for *Asparagus asparagoides*. In order to create background data in terms of the possibility that there would be fewer records returned from areas of more recent invasions and areas that were poorly sampled, we gave prominence to those having less geographical proximity to others. However, we note that without records on survey effort in terms of time, one cannot differentiate between environmentally unsuitable and undersampled areas and that these adjustments will unavoidably confuse the two categories. To calculate weighting surface, the number of weighted records (Gaussian kernel method with a standard deviation of the default values in ArcGIS) in a selected geographical environment for each cell for the whole world, with the exclusion Australia, was divided by the weighted number of terrestrial cells in the specific geographical environment (to avoid edge effects along coasts). The resulting grid was then scaled to give a maximum of 20 and a minimum of 1, to exclude extreme values (see Fig. S1). This method of weighting is advocated by Elith et al. (2010) to minimize bias favoring records from densely sampled areas over those from sparsely sampled areas. The kernel density layer of every species and the Hawth's Analysis Tools were used for generating background points for the whole world, with the exclusion of Australia, to be used for training purposes. Background points for Australia were generated, using the same method, for comparison of the model performances; see Figure S2. Thus, the performance of all SDMs was evaluated using the same background data for each species. Figure S2 displays the multivariate environmental similarity surface (MESS) maps representing the similarity of each point in the region of projection, in relation to a set of specified reference points.

### Mechanistic model

#### CLIMEX

The semimechanistic modeling method CL (Sutherst et al. 2007) studies the relationships between distributions of a species, its growth patterns, and climate (Macfadyen and Kriticos 2012). Empirically measured parameters, together with point distribution records, are used to fit models. As CL integrates generalized ecophysiological parameters, simple trait evolution may also be studied by changes in the parameters of the model, to match observations. CL may be described as a dynamic model, in that it integrates the population's weekly responses to climate into an annual indices series. The model makes the assumption that an organism's population increases during a favorable season and decreases during an unfavorable season. The CL Growth Index (GI_W_) combines data relating to temperature, moisture, and day length into a weekly index for target species. Response to general temperature and moisture conditions are expressed by the annual temperature (TI) and moisture (MI) indices. A series of stress indices (SI) incorporates responses to extreme conditions, estimating threats posed by extreme or prolonged hot, cold, wet, or dry conditions. Additionally, impositions on life cycle completion due to prolonged periods of inappropriate temperature or day length system may be analyzed, if the appropriate data are available. Lastly, an Ecoclimatic Index (EI), scaled from 0 (no persistence) to 100 (maximal population size in relation to climate alone), integrates the growth and SI as a representation of a specific geographical location's overall suitability for propagation and persistence of a species. A natural occurrence is only possible where EI is greater in value than zero (Sutherst et al. 2007; Park et al. 2014).

### Model framing in CLIMEX

As this study compares the abilities of various techniques to project suitable climate for independent records of the species in Australia, the intention in selecting species was to find species available at a global scale and in Australia, which were previously studied using CL. Scott and Batchelor (2006) provide a detailed explanation of CL parameters on *Asparagus asparagoides*, Shabani and Kotey (2016) on *Triticum aestivum* L., Taylor et al. (2012) on *Lantana camara* L., Shabani et al. (2012) on *Phoenix dactylifera* L., Pattison and Mack (2008) on *Triadica sebifera*, Shabani et al. (2014) on *Fusarium oxysporum* f. spp., Kriticos et al. (2009) on *Opuntia robusta,* and Shabani and Kotey (2016) on *Gossypium*. It should be mentioned that the CL parameters taken from other studies are based on occurrences on a global scale and Australia, while in our study, Australian occurrences were not considered. We rebuilt the CL models for each species by considering the global occurrence, excluding Australia. Thus, parameters for each species were modified to maintain an optimized CL projection for occurrence data at the global scale excluding the Australian ones, through lack of occurrence data in Australia. Refer to Table S1 for the details on values and predictors that were used for each species on CL and correlative models.

### Assessing the performance of species distribution models

To assess the performance of SDMs, we utilized the Hanssen–Kuipers discriminant or true skill statistic (TSS), which expresses Sensitivity + Specificity – 1 (Allouche et al. 2006). Sensitivity denotes the proportion of predicted observed presences, thus quantifying omission errors. Specificity denotes the proportion of predicted observed absences, thus quantifying commission errors. As an alternative, the receiver operating characteristic (ROC) curve (Fielding and Bell 1997) can be used to assess an ordinal score model's accuracy. All possible thresholds are used in the construction of ROC curves, classifying scores into confusion matrices, finding Sensitivity and Specificity for each matrix, and thereafter plotting Sensitivity against the corresponding proportion of false positives, equal to 1 − Specificity. Using all possible thresholds overrides the need for the selection of a single threshold, which may be arbitrary (Liu et al. 2005), allowing for an appreciation of the compromise between Sensitivity and Specificity (Pearce and Ferrier 2000). The area below the ROC curve (AUC) is frequently employed as a single threshold‐independent rating of model performance (Thuiller et al. 2005). AUC has been shown to be prevalence independent (McPherson et al. 2004) and is thus seen to be a highly effective indicator of ordinal score model performance. In conservation planning, however, the practical purposes of SDMs, for example, identifying biodiversity hotspots and representative conservation sites, frequently demand presence–absence maps of the distributions of species, and therefore the choice of a threshold for the transformation of ordinal results into presence–absence predictions (Berg et al. 2004). For these purposes, the evaluation of accuracy in prediction should rather be based on the selected threshold, as opposed to threshold‐independent ROC curves. Bioclim and some other more frequently used SDMs generate dichotomous presence–absence species distribution predictions, to which ROC curves cannot be applied. In this regard, research has shown AUC to be well suited to evaluating ordinal score model performance in logistic regression, in terms of its threshold‐independent nature (Allouche et al. 2006).

In this study, we divided presence points into two sample data categories of training and test points per species. The presence points of the complete species global distribution, with the exception of the Australian continent, were used as the training dataset, and the out‐of‐sample data (Australian occurrences) were used to test SDM performance. We focused on the area under the ROC curve (i.e., AUC), true statistical skill (TSS), and Sensitivity (i.e., true‐positive rate) to evaluate SDM performance. To calculate AUC, we plotted the Sensitivity of SDMs against 1 – Specificity, and to determine TSS, we calculated Sensitivity + Specificity – 1. Due to the mechanistic physiologically based manner of CL and its independency from presence‐background data, we could not calculate these metrics of SDM performance. However, we considered Sensitivity and fractional predicted area of Australia (the proportion of cells predicted to have suitable habitat for the species (Phillips et al. 2006)) to compare the performance of all SDMs, including the CL model. As we had no data on species true absence in Australia for each model, we calculated the proportion of the extent of Australia identified as suitable, as an index of model's potential overestimation. To identify the suitable range from unsuitable climatic extent, particularly used for calculation of threshold‐dependent metrics of evaluation (e.g., Sensitivity, Specificity, and TSS), we employed the minimum training probability of presence as the presence/absence threshold.

### Assessing the satisfactory agreement between the output of six different bioclimatic models on eight species in Australia (spatial comparison)

There were 720 possible comparisons (combinations) to assess the extent of agreement between the output of six different bioclimatic models and eight species (spatial comparison); however, in this study, we compared the outputs of the mechanistic model against the correlative models. Thus, CL outputs on the eight species were extracted using ArcGIS software. All areas with EI > 0, for each species, were overlaid onto suitable areas projected separately by GLM, MaxEnt, Bioclim, RF, and BRT. Thereafter, all locations that satisfied the conditions of suitability for each correlative model, together with CL, were selected and extracted on the basis of agreement between models (CL with GLM, CL with MaxEnt, CL with Bioclim, CL with RF, and CL with BRT).

## Results

**Figure 1:**
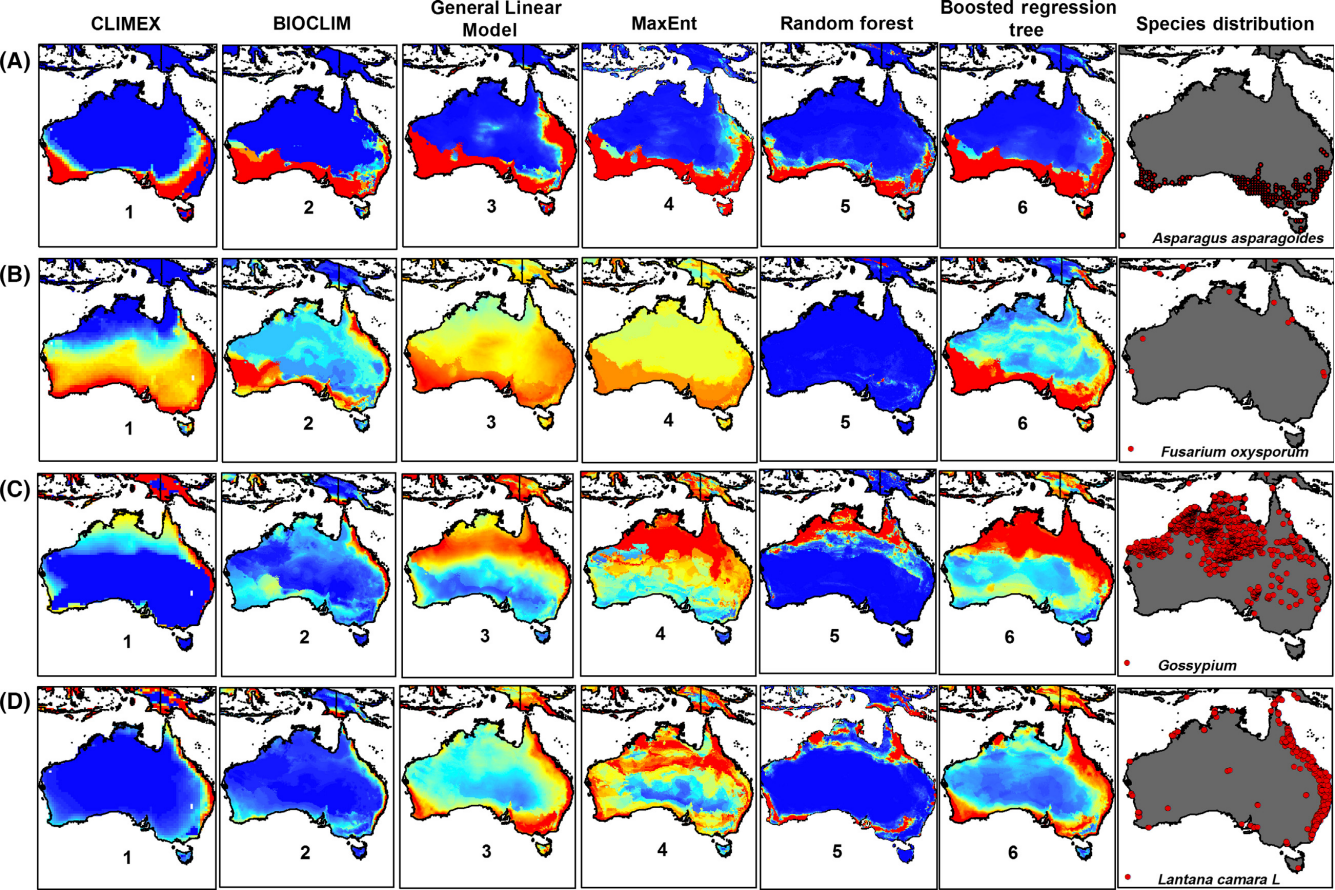
Projections of an independent area for the potential distribution of (A) *Asparagus asparagoides*, (B) *Fusarium oxysporum* f. spp., (C) *Gossypium*, and (D) *Lantana camara* L. using correlative and mechanistic niche models. Warmer colors show areas with better‐predicted conditions.

**Figure 2:**
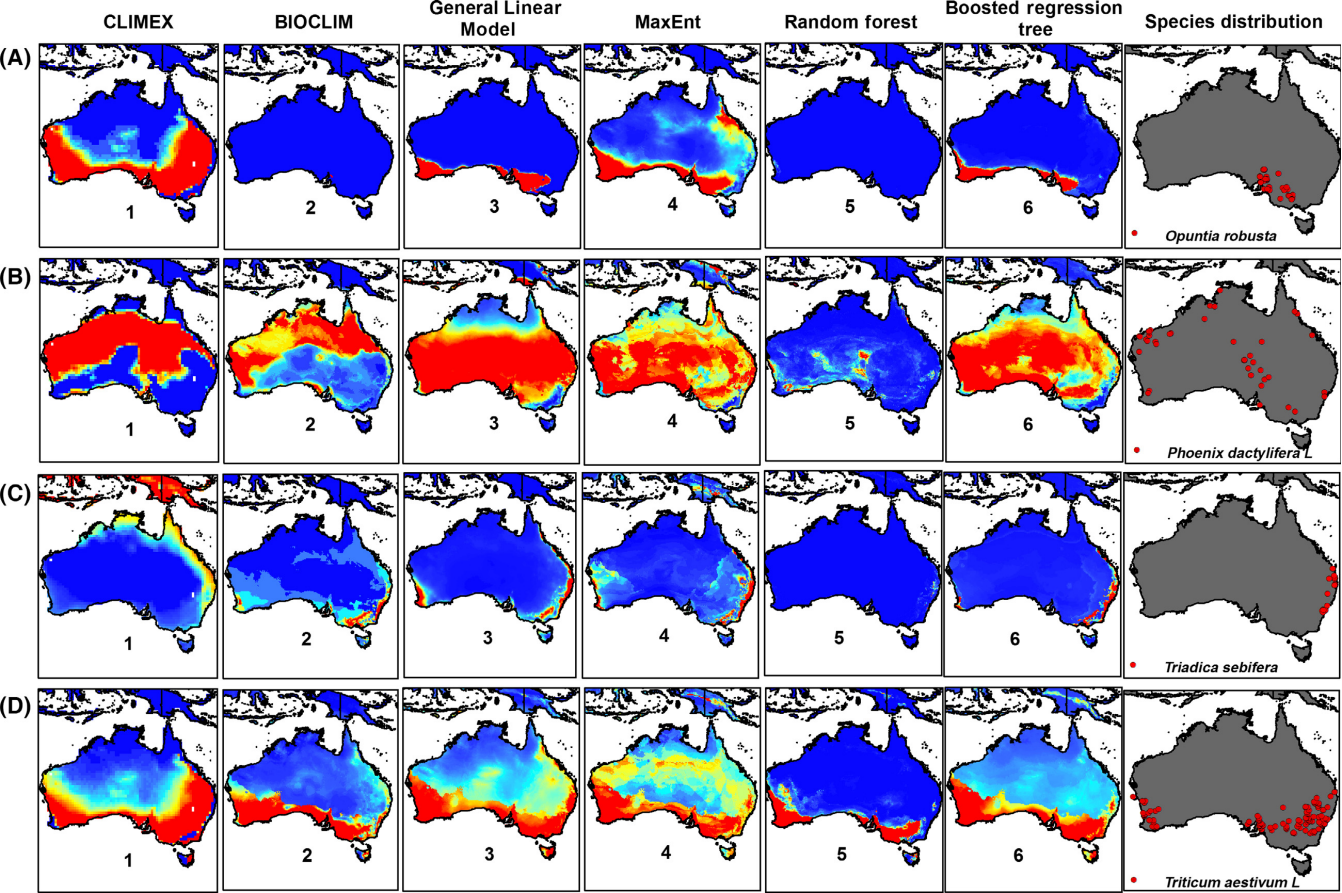
Projections of an independent area for the potential distribution of (A) *Opuntia robusta*, (B) *Phoenix dactylifera* L., (C) *Triadica sebifera*, and (D) *Triticum aestivum* L. using correlative and mechanistic niche models. Warmer colors show areas with better‐predicted conditions.

The modeling method showed an impact on predicted potential distributions for an independent area, and there were differences between various method projections (Figs 1 and 2). Our results indicated that the mean AUC values, achieved by the five correlative models on eight species, were above 0.77. Despite having a high AUC value, the model based on the training dataset failed to predict the occurrence of the studied species in some places where it is clearly known to occur (Figs 1 and 2).

Comparing TSS of different models, based on test data (Australian occurrences), shows the relatively comparable measurement of the modeled TSS between the Bioclim, GLM, and MaxEnt models for *Asparagus asparagoides*,* Phoenix dactylifera* L., and *Gossypium,* (Table 2). For example, TSS for *Asparagus asparagoides* in Bioclim, GLM, MaxEnt, and BRT models were 0.73, 0.72, 0.74, and 0.76, respectively, while only 0.42 in RF model. A similar trend was also seen for *Lantana camara* L. (Table 2). In contrast, in the related comparison for *Triadica sebifera*, a moderate correlation between TSS of each model was achieved; TSS for *Triadica sebifera* from Bioclim, GLM, MaxEnt, BRT, and RF were 0.85, 0.90, 0.27, 0.29, and 0, respectively.

**Table 2:** AUC and true skill statistic (TSS) of the different models for eight species for training dataset (all the world, excluding Australia) and an independent area (Australia). Nonbold values are for training dataset and the bold values are for an independent area

	Bioclim				GLM				MaxEnt				BRT				RF			
	AUC	**AUC**	TSS	**TSS**	AUC	**AUC**	TSS	**TSS**	AUC	**AUC**	TSS	**TSS**	AUC	**AUC**	TSS	**TSS**	AUC	**AUC**	TSS	**TSS**
*Asparagus asparagoides*	0.897	**0.936**	**0.622**	**0.734**	0.930	**0.950**	0.747	**0.725**	0.960	**0.957**	0.799	**0.742**	0.957	**0.954**	0.824	**0.765**	1	**0.929**	0.980	**0.424**
*Phoenix dactylifera* L.	0.780	**0.610**	**0.413**	**0**	0.823	**0.520**	0.487	**0.1**	0.894	**0.520**	0.626	**0**	0.907	**0.517**	0.666	**0**	1	**0.574**	0.989	**0**
*Fusarium oxysporum* f. spp.	0.630	**0.676**	0.210	**0.283**	0.671	**0.571**	0.268	**0.184**	0.725	**0.679**	0.342	**0.211**	0.857	**0.612**	0.558	**0.259**	1	**0.584**	0.997	**0**
*Gossypium*	0.709	**0.560**	**0.282**	**0**	0.754	**0.669**	0.424	**0.201**	0.826	**0.725**	0.495	**0.380**	0.827	**0.717**	0.504	**0.359**	1	**0.756**	0.986	**0.135**
*Lantana camara* L	0.710	**0.984**	**0.276**	**0.663**	0.740	**0.988**	0.375	**0.846**	0.812	**0.876**	0.475	**0.606**	0.813	**0.973**	0.488	**0.869**	1	**0.757**	0.982	**0.164**
*Opuntia robusta*	0.967	**0.552**	**0.931**	**0**	0.967	**0.849**	0.932	**0.575**	0.970	**0.907**	0.872	**0.461**	0.987	**0.906**	0.962	**0.015**	1	**0.507**	0.998	**0**
*Triadica sebifera*	0.865	**0.935**	**0.665**	**0.853**	0.886	**0.989**	0.715	**0.901**	0.934	**0.980**	0.746	**0.273**	0.927	**0.981**	0.750	**0.293**	1	**0.981**	0.989	**0**
*Triticum aestivum* L.	0.688	**0.790**	**0.270**	**0.378**	0.745	**0.802**	0.381	**0.410**	0.796	**0.761**	0.453	**0.293**	0.794	**0.791**	0.467	**0.296**	1	**0.648**	0.967	**0.239**
Mean	0.781	**0.755**	0.459	**0.364**	0.815	**0.792**	0.541	**0.493**	0.865	**0.801**	0.601	**0.371**	0.884	**0.806**	0.652	**0.357**	1	**0.717**	0.986	**0.120**
SD	0.110	**0.167**	0.255	**0.352**	0.104	**0.187**	0.230	**0.315**	0.088	**0.158**	0.189	**0.233**	0.071	**0.177**	0.180	**0.314**	0	**0.171**	0.010	**0.154**

BRT, Boosted Regression Tree; GLM, Generalized Linear Model; RF, Random Forest.

Comparing the mean TSS of the eight species shows that GLM had the highest TSS (0.49), while Bioclim, MaxEnt, BRT, and RF values were 0.36, 0.37, 0.35, and 0.12, respectively. In this comparison, the lowest TSS belonged to RF, which indicates the poorest performance of the model in extrapolation, when compared to the performances of Bioclim, GLM, MaxEnt, and BRT (Table 2).

Comparing the mean Sensitivity of different correlative models for the eight species shows that Bioclim, GLM, and MaxEnt had a similar and consistent Sensitivity (0.78, 0.86, 0.84), when compared to BRT and RF (0.67 and 0.035).

**Figure 3:**
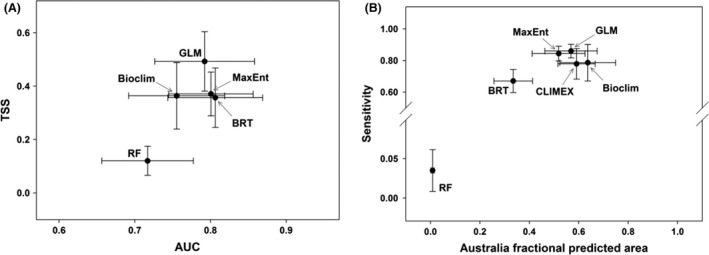
(A) True skill statistic and AUC in correlative models for eight species using evaluation data. (B) The Sensitivity of correlative and mechanistic models with fractional predicted areas in Australia.

The five correlative modeling techniques applied here differ in how they deal with extrapolation (Figs 1 and 2). The evaluation of correlative and envelope SDMs performance, based on mean TSS and mean AUC values among eight species, showed that GLM and MaxEnt had TSS of 0.41 and 0.29 with AUC of 0.80 and 0.76 (Fig. 3A), while TSS and AUC for other SDMs were close to, but lower than the GLM and MaxEnt values.

As mentioned earlier, because of the mechanistic physiologically based manner of CLIMEX and its independency from presence‐background data, we could not calculate TSS and Specificity metrics on this model. However, we considered Sensitivity and fractional predicted area in Australia to compare the performance of all SDMs, including CLIMEX. The comparison of models' Sensitivity against fractional predicted area in Australia showed that GLM and MaxEnt, with the best Sensitivity, calculated a relatively low fractional predicted area of Australia, compared to other SDMs, revealing a low probability of overestimation of this model in an independent area. We found that Bioclim computed the largest proportion of an independent area as suitable climate condition (i.e., fractional predicted area) (Fig. 3B). GLM, MaxEnt, and CL had an acceptable and close Sensitivity and fractional predicted area, while BRT and RF had the poorest performance (Fig. 3B).

**Figure 4:**
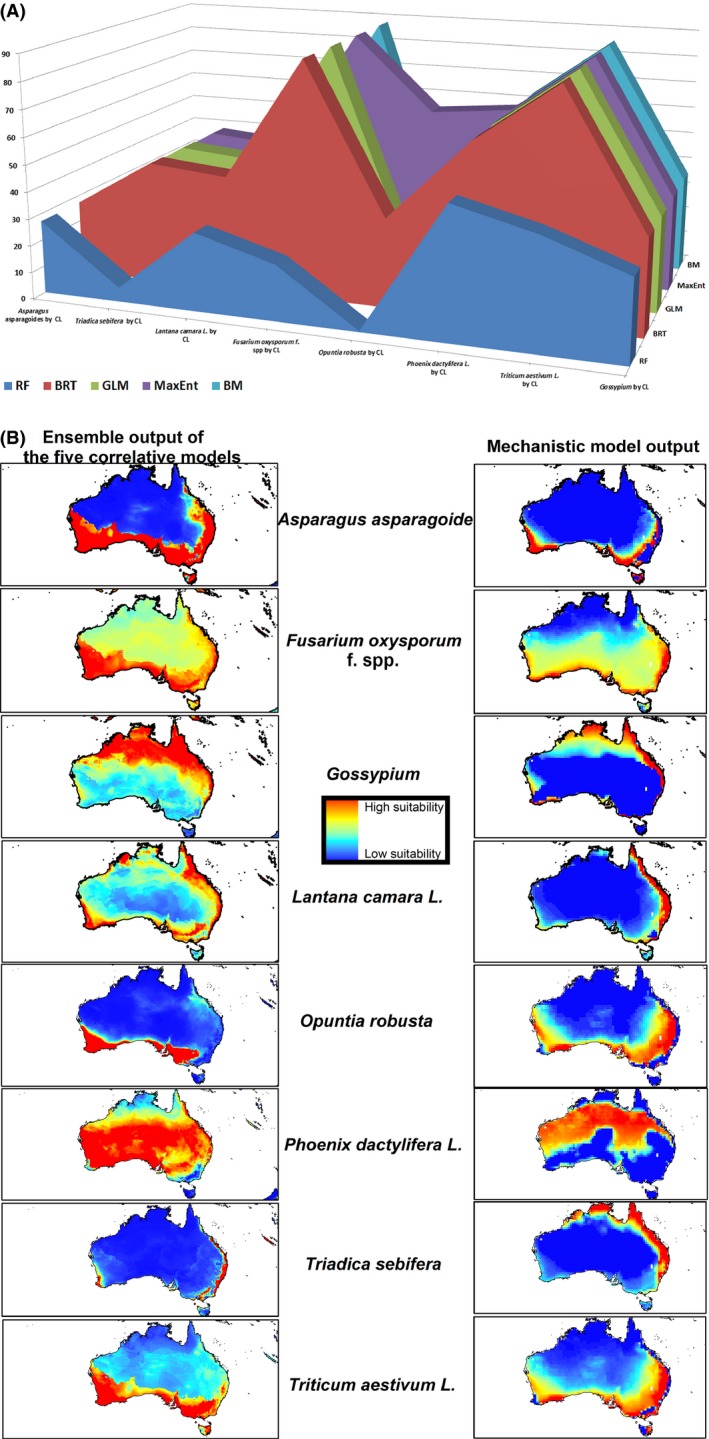
(A) Spatial comparison on a satisfactory agreement between the output of five correlative models with CLIMEX (CL), using eight species (B) comparing the individual technique of CL to an ensemble approach of the five correlative models.

Spatial comparison on satisfactory agreement between the output of six different bioclimatic models on *Triticum aestivum* L. and *Fusarium oxysporum* f. spp. showed that Bioclim, GLM, MaxEnt, and BRT were 85–90% in agreement with CL extrapolation outputs, while RF's outputs were about 30% similar to CL projections spatially (Fig. 4A). Similar comparisons for *Triadica sebifera* showed that there was an agreement between BIOCLIM, GLM, MaxEnt, and BRT projections with CL outputs (Fig. 4A). In contrast, differences on CL, Bioclim, GLM, MaxEnt, and RF extrapolation outputs for *Opuntia robusta* were significant (Figs 2A and 4A). Figure 4A also indicates that 30–35% Bioclim, GLM, MaxEnt, and BRT's outputs were similar to CL projections. On the other hand, the comparison of the individual technique of CLIMEX to an ensemble approach of the five correlative models (Fig. 4B) for eight different species showed that there was a better agreement between the ensemble output of correlative model projections with CL outputs (Fig. 4B) when compared to only using single‐modeling techniques (Figs 1, 2, and 4A).

## Discussion

In this study, data were obtained from various databases, while presence data were collected in the field and can therefore represent local (microclimate) suitable for the species, although not reflected by the coarse 10' climate data. This might lead to overly optimistic predictions regarding suitable climate, particularly as some of the calibration areas might have a greater variety of climatic conditions within a 10' pixel, than in Australia (Austin and Van Niel 2011). However, we were limited by the data available at a global scale for the modeling, of which the best available is 10' climate data. Microscale data would have been ideal, but were not available.

A number of the plant species modeled are invasive species (*Asparagus asparagoides*,* Lantana camara* L., *Opuntia robusta*) in Australia, which may have implications for evaluating model fit, as these species may not yet have filled their potential distributions and models appearing to overestimate distribution limitations may not be fully reflective. Alternatively, other species modeled are cultivated (e.g., *Triticum aestivum* L., *Phoenix dactylifera* L., *Gossypium*), which also may have implications for the model evaluation, as factors aside from climate may determine where these species are grown (Shabani and Kumar 2014; Shabani et al. 2016). However, distinguishing between exotic and native records was outside the scope of the study and thus this is one of the limitations.

Analysing the results displayed in Figures 1 and 2 gives rise to a range of questions concerning characteristics required from models in specific situations. For example, in terms of the use of SDMs for predicting species range shifts under climate change, or the extrapolation of their movement to new regions, it is essential to understand the performance of the algorithm, as projected for environmental combinations not sampled by the training data. Thus, the question of whether the algorithm extrapolation is appropriate from an ecological perspective must be asked. In testing the implementation of the various methods, different behaviors were apparent; however, the choice of which is most appropriate should be seen to be as much an ecological and/or physiological question as a statistical one. On the operational level, there were more choices than those which we demonstrated – for example, MaxEnt has inbuilt options for predicting absence, beyond the data range. A far larger range of tests is demanded, including prediction to new combinations of environments, for a complete investigation of extrapolation or forecasting behavior. It is paramount to first understand the different modeling needs relevant to specific SDMs and thereafter research the best means of achieving these requirements. Understanding the workings of specific models and the devising of evaluation criteria closely matched to the associated questions enable best decisions regarding the modeling approach (Elith & Graham, 2009). For more clarification, refer to Elith and Graham (2009).

### Why not AUC?

Species distribution models are valuable tools in addressing questions and issues in the fields of climate change ecology, and biogeography, as well as in evolutionary and conservation biology, and thus, understanding performance testing and evaluation methods of correlative and mechanistic models is vital to their practical usefulness (Guisan and Thuiller 2005). In this respect, AUC is a frequently used measure of model performance (Manel et al. 2001; Thuiller et al. 2005; Lobo et al. 2008), having been shown to be independent of prevalence, in both the theoretical (Hanley and McNeil 1982; Zweig and Campbell 1993) and empirical spheres (McPherson et al. 2004). In measuring model performance, AUC is threshold independent and thus particularly suitable for the performance evaluation of ordinal score models such as logistic regression with true presence–absence data. But most of the time, data of the absence locations are not available and presence data are the only accessible data of the species. In this situation, envelope (e.g., Bioclim) or distance‐based (e.g., Domain or Mahalanobis distance) models are the option for SDM (Farber and Kadmon 2003). However, in practice, a dichotomous prediction of presence–absence is frequently demanded, thus necessitating the application of a threshold to transform probability/suitability scores into presence–absence data. For example, presence–absence data on species composition in a specific location are required for most reverse selection algorithms (Tsuji and Tsubaki 2004). As data available are often incomplete, SDMs are frequently employed for predicting the presence or absence of a particular species in a potential locality (Sánchez‐Cordero et al. 2005). Estimating the hotspots of biodiversity is also frequently presence–absence prediction based (Schmidt et al. 2005). Assessing global change impacts at the community levels could be achieved by species assemblage prediction of stacked binary SDMs (Guisan and Rahbek 2011; D'Amen et al. 2015). Presence–absence predictions do not allow for the construction of ROC plots and, thus, AUC cannot be used to evaluate accuracy of the predictive maps used in this type of application. Results shown in Table 2 indicate that a high value of AUC for each species and for each model does not guarantee the output accuracy. In this regard, we believe that the MESS maps will not identify changes in correlations between variables, and tests for these are also critical because the model parameters are estimated on the correlation structure between predictors in the training data. For most models, predictions to areas with substantially different correlations between important variables will be unreliable (Harrell 2001 2015). This is particularly problematic when the available predictors are only indirectly related to the species' distribution (Austin 2002). The selected set might together represent the unmeasured directly influential variable reasonably well, but if correlations between them change in new areas, prediction will be compromised.

Utilizing TSS measurement of accuracy, which is insensitive to prevalence (Allouche et al. 2006) and the fractional predicted areas (Phillips et al. 2006) in Australia of eight different species, showed that GLM and MaxEnt had the best Sensitivity, while Bioclim was the best on fractional predicted area (Fig. 3B). Bioclim and CL outputs were also close to GLM and MaxEnt and, therefore, could be considered in the same cluster (Fig. 3B). We also note that the comparison of the individual technique of CL to an ensemble approach of the five correlative models showed that there was a better agreement between the ensemble output of correlative model projections with the mechanistic model output when compared to only using single‐modeling techniques. This finding is in line with Araújo and New (2007) who have documented that using ensemble forecasting has clear advantages over single model forecasts. In this regard, it should be noted that our results also indicated that it is paramount to have some knowledge of how reliable SDM predictions are and that ideally this should be tested on an individual case basis, as the TSS for *Asparagus asparagoides* in Bioclim, GLM, MaxEnt, and BRT models was 0.73, 0.72, 0.74, and 0.76, respectively, while it was 0.42 in RF model. In addition, the Sensitivity of *Asparagus asparagoides* in Bioclim, GLM, MaxEnt, and BRT models was 0.98, 0.96, 0.98, and 0.95, respectively, while it was 0 in RF. In contrast, AUC for this species was 0.93, 0.95, 0.95, 0.95, and 1 in Bioclim, GLM, MaxEnt, BRT, and RF, respectively (Table 2); this finding agrees with Lobo et al. (2008) who documented that AUC is not an appropriate measure of comparative accuracy between model results for five reasons: (1) the probability values predicted and the closeness of fit of the model are ignored; (2) the test performance summary includes regions of the ROC space which would rarely be operative; (3) commission and omission errors are equally weighted; (4) there is no information about the model errors' spatial distribution; and, most importantly, (5) the extent to which modeling is executed strongly influences the well‐predicted absence rate and the AUC scores. This, however, does not imply that any of these statistics are misleading (Lobo et al. 2008) but simply that different aspects of performance are measured by different statistics and that decisions must be made regarding the relevance to the application of the model in terms of appropriate statistics.

It should be noted that there are a number of important decisions to be made in constructing an SDM and our study, as well as other related studies, describes factors which can impact on or limit results including (1) occurrence in theoretically unsuitable habitat of a particular mobile species, (3) occurrence in theoretically unsuitable habitat of sessile species (e.g., plants), (3) failure to observe a species in a suitable habitat, (4) low detectability of a particular species, (5) ecotypes of the same species and sibling species, (6) historical bias in natural history collections, and (7) no absences (Guisan and Thuiller 2005; Václavík and Meentemeyer 2012).

We also note that in the present study, geographic location and “space” were not taken into account and that therefore results may have been different if dispersal, barrier effects, and biogeographic history had been included. Australia might indeed have areas with climate combinations that are found nowhere else on the planet (calibration data) and therefore represent a “an independent area”/no‐analog climate. Finally, we highlight that in this study, the absolute performances of different models in the “an independent area” climate were investigated. Differences in model performance of “known” and “in an independent area” climate for the different techniques were also observed; some had a higher AUC, TSS, but a large difference in performance between “known” and “an independent area” climate, while others had a lower AUC, TSS but more stable performance in different climate spaces (Engler and Guisan 2009). Thus, it might be useful to consider the geographic locations and “space.”

## Conclusion

Understanding the application of a particular algorithm gives insight into various features related to its predictions, helping to answer why particular patterns occur. As each one of these models (CL, Bioclim, GLM, MaxEnt, BRT, and RF) provides slightly different results on projections, it may be safer to use an ensemble of models. Utilizing the impact of measurement of accuracy, TSS, and the fractional predicted areas on eight different species in Australia showed that Bioclim, GLM, MaxEnt, and CL outputs were generally close and produced a better performance in comparison with BRT and RF.

With conservation strategies becoming increasingly reliant on the distribution model outputs, it is essential to understand the level of accuracy of output predictions, which should be tested on an individual case‐by‐case basis. Where insufficient data impede validation, a rough guide to factors impacting on model reliability fulfills the purpose. Model reliability is dependent on both the data properties used in parameterization and the spectrum of ecological characteristics of a particular species. However, as the ecological characteristics impact on our ability and the possibilities to collect the necessary data, methodological and ecological factors affecting output accuracy are frequently intertwined.

Future improvements to methodology include comparative assessment of how various scales may be considered in SDMs, in relation to species behavioral characteristics, dispersal ability, study area extent and parameters, and general data characteristics, and enhancing evaluation and validation frameworks for assessing SDM errors and inaccuracies.

## Conflict of Interest

None declared.

## Supporting information


**Figure S1.** The kernel density map of *Asparagus asparagoides* and *Gossypium* that were used to generate background points for the training purposes and the background points generated for Australia for model comparison.
**Figure S2.** The multivariate environmental similarity surface (MESS) maps of all the eight species.
**Table S1**. CLIMEX parameter values as obtained from the literature for the various species to model the global distribution.
**Table S2.** Comparison of AUC, TSS and performance of the different models for *Lantana camara* L for the known and novel environments.
