# Supplementary material for: A comparison of absolute performance of different correlative and mechanistic species distribution models in an independent area
Source: Ecol Evol. 2016 Jul 27;6(16):5973–86. doi: 10.1002/ece3.2332 (PMC4983607; doi:10.1002/ece3.2332)
Supplement: Supplementary file 1 — Figure S1. The kernel density map of Asparagus asparagoides and Gossypium that were used to generate background points for the training purposes and the background points generated for Australia for model comparison. Figure S2. The multivariate environmental similarity surface (MESS) maps of all the eight species. Table S1. CLIMEX parameter values as obtained from the literature for the various species to model the global distribution. Table S2. Comparison of AUC, TSS and performance of the different models for Lantana camara L for the known and novel environments. [file ECE3-6-5973-s001.docx]

**Supplementary Figures**


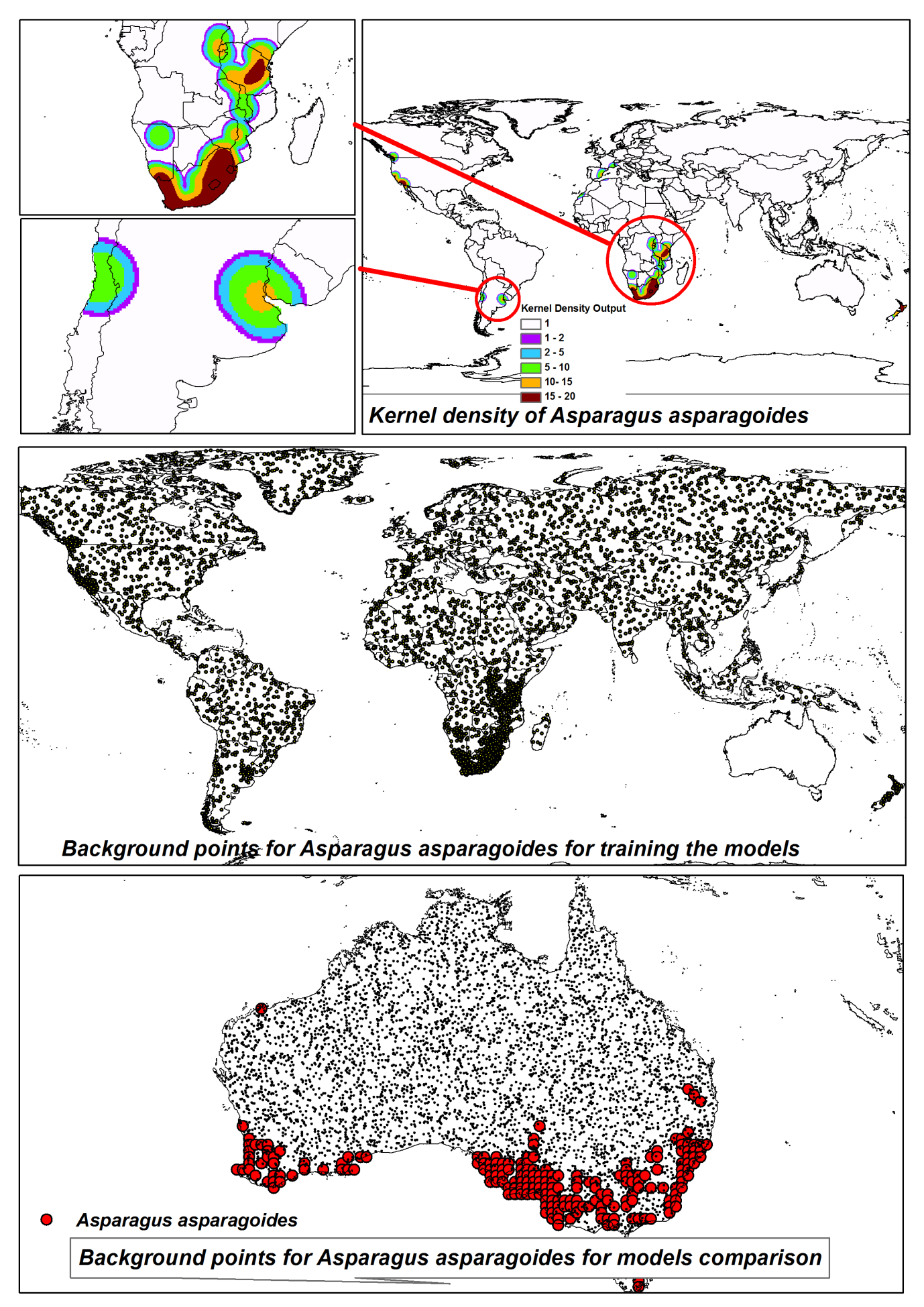


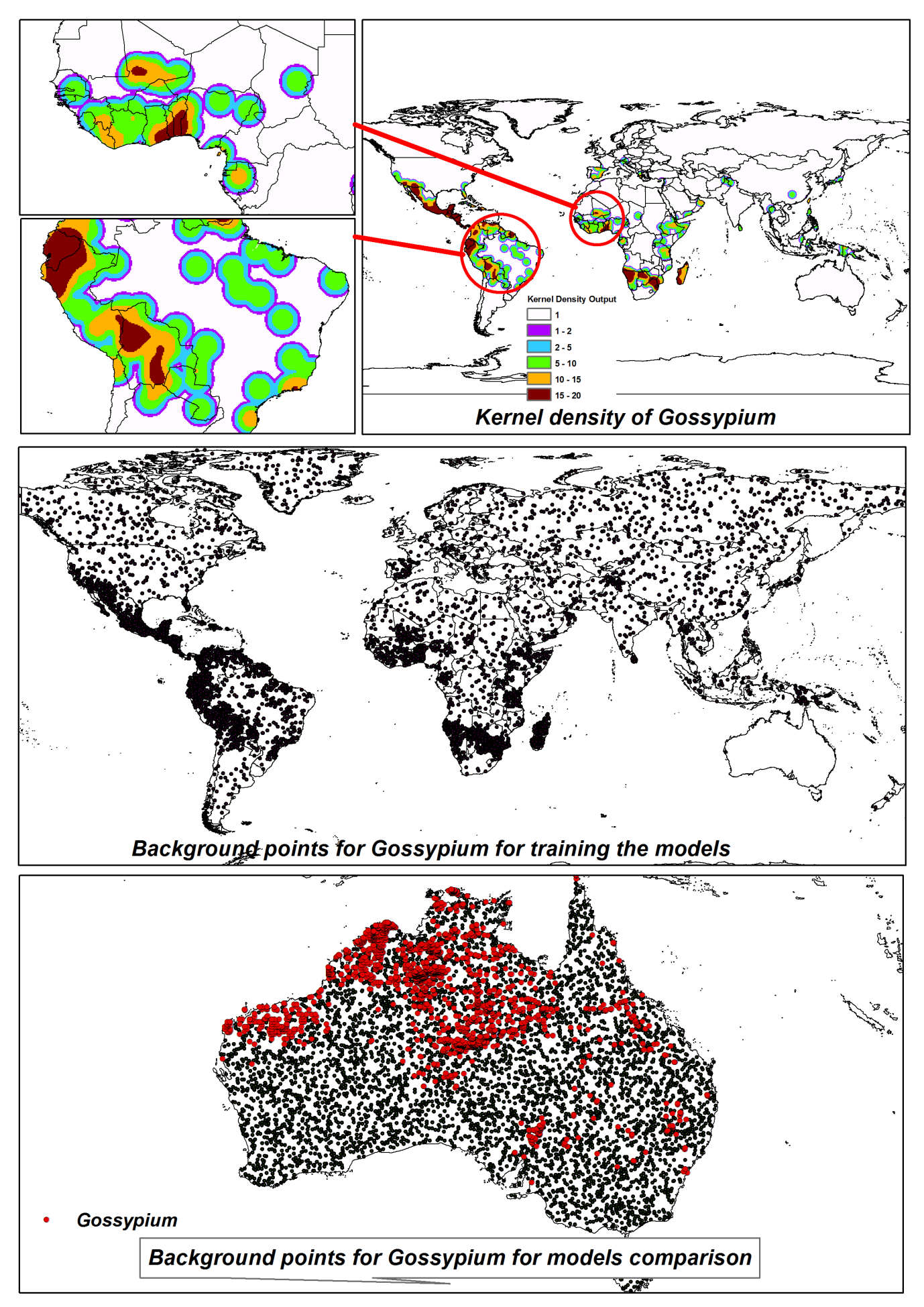
 Figure S1- The kernel density map of *Asparagus asparagoides* and *Gossypium* that were used to generate background points for the training purposes and the background points generated for Australia for model comparison.


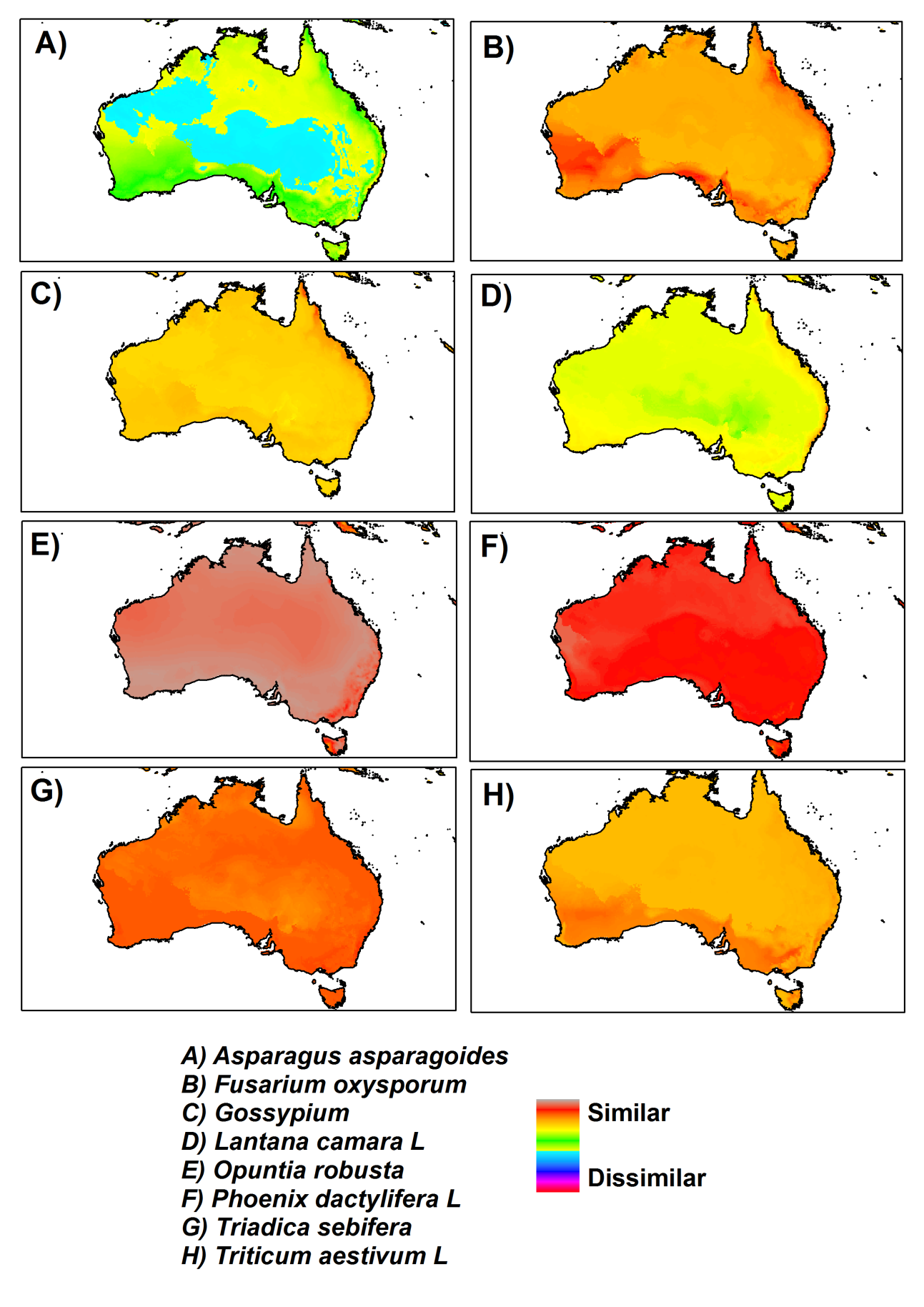


Figure S2- The multivariate environmental similarity surface (MESS) maps of all the eight species.

Table S3- CLIMEX parameter values as obtained from the literature for the various species to model the global distribution. Some of these values were refined further to ensure a minimum of 95% the training dataset fell in the suitable regions.

| Parameter | Mnemonic | Units | *Asparagus asparagoides* | [*Phoenix dactylifera*](http://journals.plos.org/plosone/article?id=10.1371/journal.pone.0083404) L. | *Fusarium oxysporum* f. spp. | *Gossypium* | *Lantana camara* L. | *Opuntia robusta* | *Triadica sebifera* | *Triticum aestivum* L. |
| --- | --- | --- | --- | --- | --- | --- | --- | --- | --- | --- |
| Limiting low temperature | DV0 | °C | 10 | 14 | 16 | 0.0001 | 10 | 8 | 12 | 0.01 |
| Lower optimal temperature | DV1 | °C | 15 | 20 | 17 | 0.3 | 25 | 18 | 24 | 0.2 |
| Upper optimal temperature | DV2 | °C | 18 | 39 | 25 | 1.9 | 30 | 24 | 35 | 0.4 |
| Limiting high temperature | DV3 | °C | 30 | 46 | 28 | 3 | 33 | 28 | 40 | 3 |
| Limiting low soil moisture | SM0 | - | 0.15 | 0.007 | 10^-5^ | 10 | 0.1 | 0.05 | 0.125 | 4 |
| Lower optimal soil moisture | SM1 | - | 0.5 | 0.013 | 0.008 | 14 | 0.5 | 0.2 | 0.25 | 14 |
| Upper optimal soil moisture | SM2 | - | 1.2 | 0.81 | 8 | 38 | 1.2 | 0.5 | 2 | 25 |
| Limiting high soil moisture | SM3 | - | 1.5 | 0.9 | 9 | 40 | 1.6 | 1 | 3 | 32 |
| Cold stress temperature threshold | TTCS | °C | 2.1 | 4 | 0.0001 | 3 | 5 | 0 | -3 | -10 |
| Cold stress temperature rate | THCS | Week ^-1^ | -0.1 | -0.01 | -0.001 | -0.1 | -0.004 | -0.01 | -0.007 | -0.001 |
| Heat stress temperature threshold | TTHS | °C | - | 46 | 28 | 40 | 33 | 32 | 42 | 39 |
| Wet stress threshold | SMWS | - | - | 0.9 | 9 | 3 | 1.6 | 1 | - | 3 |
| Wet stress rate | HWS | Week ^-1^ | - | 0.022 | 0.001 | 0.001 | 0.01 | 0.02 | - | 0 |
| Heat stress accumulation rate | THHS | Week ^-1^ | - | 0.9 | 10^-6^ | 0.001 | 0.001 | 0.005 | 0.005 | 0.005 |
| Hot-wet temperature threshold | TTHW | °C | 20 | - | - | - |  | 21 | - | - |
| Hot-wet moisture threshold | MTHW | - | 1.2 | - | - | - |  | 0.7 | - | - |
| Heat – wet stress rate | PHW | Week ^-1^ | 0.5 | - | - | - |  | 0.01 | - | - |
| Dry stress threshold | SMDS |  | - | - | 0.007 | - | 0.1 | 0.05 | - | - |
| Dry stress rate | HDS | Week ^-1^ | - | - | -0.009 | - | -0.01 | -0.005 | - | - |
| Minimum degree-day cold stress threshold | DTCS | °C days | - | - | - | - | 15 | 30 | - | - |
| Degree-day cold stress rate | DHCS | Week ^-1^ |  |  |  |  | -0.0022 | -0.0001 | - |  |
| Average maximum weekly temperature above which ‘heat and dry stress’ accumulates | TTHD | °C | - | - | - | - | - | - | 13 | - |
| Average weekly soil moisture level below which ‘heat and dry stress’ accumulates | MTHD | - | - | - | - | - | - | - | 0.125 | - |
| Rate at which ‘heat and dry stress’ accumulates | PHD | Week ^-1^ | - | - | - | - | - | - | 0.002 | - |

Table S4 - Comparison of AUC, TSS and performance of the different models for *Lantana camara* L for the known and novel environments. Known environment means that there are occurrence records in specific regions while novel environments in this study means that there are no occurrence records in specific regions. Non-bold values are for known environments (Madagascar) and the bold values are for novel environments (Libya).

| *Lantana camara* L | **Bioclim** | | **GLM** | | **MaxEnt** | | **BRT** | | **RF** | |
| --- | --- | --- | --- | --- | --- | --- | --- | --- | --- | --- |
| **Bioclim** | **-** | | 63% | **0%** | 72% | **0%** | 57% | **0%** | 19% | **0%** |
| **GLM** | 95% | **0%** | **-** | | 97% | **48%** | 98% | **38%** | 17% | **17%** |
| **MaxEnt** | 89% | **0%** | 97% | **75%** | **-** | | 97% | **17%** | 29% | **23%** |
| **BRT** | 87% | **0%** | 95% | **42%** | 93% | **27%** | **-** | | 20% | **93%** |
| **RF** | 90% | **0%** | 39% | **23%** | 52% | **16%** | 39% | **89%** | **-** | |
